# Basic and clinical assessment of initial distribution volume of glucose in hemodynamically stable pediatric intensive care patients

**DOI:** 10.1186/s40560-014-0059-y

**Published:** 2014-11-12

**Authors:** Hironori Ishihara, Eiji Hashiba, Hirobumi Okawa, Junichi Saito, Toshinori Kasai, Toshihito Tsubo

**Affiliations:** Department of Anesthesiology, Kuroishi-Kousei Hospital, 9-1 Tateishi, Kuroishi-shi, Aomori 036-0351 Japan; Department of Anesthesiology, Hirosaki University Graduate School of Medicine, 5 Zaifu-cho, Hirosaki-shi, Aomori 036-8562 Japan

**Keywords:** Distribution volume, Glucose, Measurement techniques, Children, Cardiac output

## Abstract

**Background:**

Initial distribution volume of glucose (IDVG), which is not associated with significant modification of glucose metabolism, has been proposed as an indicator of the central extracellular fluid volume status in adults. However, data on IDVG in children are lacking. This study examined pharmacokinetic data on IDVG in children and compared IDVG with other clinical variables.

**Methods:**

In total, 128 daily data sets from 60 consecutive pediatric intensive care patients (body weight ≥8.0 kg), consisting mostly of children undergoing cardiovascular surgery, were studied. Either 1 or 2 g of glucose based on body weight (approximately 0.1 g/kg) was administered. IDVG could not be determined from ten data sets from eight children because of body movement-associated glucose fluctuation during measurement. In the remaining 113 data sets from 55 children, IDVG was determined by applying the one-compartment model. Approximated IDVG based on the incremental plasma glucose level at 3 min postinjection (1-point IDVG), and approximated IDVG based on incremental plasma glucose levels at 3 and 5 min postinjection (2-point IDVG), were also calculated. Postoperative daily IDVG and the relationship between IDVG and cardiac output or circulating blood volume (CBV) were evaluated when data were available.

**Results:**

Convergence was assumed in each glucose clearance curve. Mean indexed IDVG (IDVGI) of the first measurement in 55 children was 144 ± 22 (SD) mL/kg, which was associated with a plasma glucose disappearance rate (Ke-glucose) of 0.094 ± 0.033/min. Bias and precision were smaller between 2-point IDVG and standard IDVG than between 1-point IDVG and standard IDVG (−0.02 ± 0.13 L versus 0.07 ± 0.20 L, *p* <0.001). Postoperative IDVGI in 37 children after cardiovascular surgery increased daily on postoperative days 1–2 (*p* ≤0.011). Linear correlations were observed between IDVGI and indexed cardiac output (*r* = 0.588, *n* = 28, *p* <0.001) and between IDVGI and indexed CBV (*r* = 0.547, *n* = 25, *p* = 0.0047).

**Conclusions:**

IDVG is a potential marker of fluid volume status in children, even though body movement-associated glucose fluctuation is a major limitation. Two-point IDVG is preferable to 1-point IDVG for approximated IDVG.

## Background

Fluid volume management is a daily challenge in critically ill children, since static preload variables such as central venous pressure (CVP) have been shown to be of limited value in children [1,2]. Dynamic preload variables such as stroke volume variation which reflect ventilation-induced cyclic changes in left ventricular stroke volume have been shown to be reliable in adults, but are not consistent in children, since the high chest wall, lung, and arterial vascular compliance in children may reduce the impact of ventilation on the cyclic changes [2]. Furthermore, the use of an esophageal Doppler is accurate in pediatric patients only if used by a skilled operator [3]. This may limit its utility in routine clinical practice.

The initial distribution volume of glucose (IDVG), which is not associated with significant modification of glucose metabolism, has been proposed to be representative of the central extracellular fluid (ECF) volume status [4-6]. It can be measured simply and rapidly in any intensive care unit (ICU) by injecting a small amount of glucose (5 g) in adults and determining the changes in plasma glucose at 3 min postinjection [7]. Measurements can also be repeated at 30-min intervals, and sustained increases in plasma glucose do not occur [8]. We previously reported that IDVG, rather than intrathoracic blood volume or CVP, correlated closely with cardiac output (CO) during hypotension and subsequent fluid volume loading in the early postoperative period after esophagectomy [9]. Moreover, DVG was reported to be a predictor of hypovolemic hypotension in the early postoperative period after abdominal aortic surgery [10] and had an inverse correlation with pulse pressure variation and the pleth variability index [11,12]. Accordingly, IDVG has the potential to be a useful marker in fluid volume management in adults. However, there have been no reports regarding IDVG in children.

This study aimed to examine pharmacokinetic data on IDVG and to compare IDVG with other clinical variables in hemodynamically stable pediatric intensive care patients.

## Methods

Ethical approval for this study was given by the Ethical Committee of Hirosaki University Graduate School of Medicine, Hirosaki, Japan. Parents gave written informed consent before admission to the ICU. Eligible children were those with a body weight ≥8.0 kg who had a radial artery line and a central venous line for routine cardiovascular management during their stay in the ICU. Exclusion criteria for IDVG determination include the presence of hyperglycemia (>200 mg/dL) and central nervous system ischemia. A total of 128 data sets from 60 children were obtained. Most children underwent surgery for congenital heart disease and were admitted to the ICU postoperatively on the operative day.

### Measurement procedures

Initial measurements were taken once per day during the stay in the ICU. After confirming that the patient had a relatively stable hemodynamic state, 2 mL of 50% glucose solution (Otsuka Pharmaceutical Inc., Japan) (1 g) diluted with 2 mL of additional normal saline solution (total of 4 mL) was injected through the central venous line to calculate IDVG. Blood samples were obtained through the radial artery catheter immediately before and at 3, 4, 5, 6, and 7 min after the injection. The total amount of sampled blood was <2.5 mL for each IDVG determination, which included arterial blood gas analysis and complete blood count. Plasma was separated immediately, and measurements of glucose concentrations were performed within 5 min of sampling. Plasma glucose concentrations were measured using amperometry by a glucose oxidase-immobilized membrane—H_2_O_2_ electrode (glucose analyzer GA-1150; Arkray Co., Ltd., Kyoto, Japan). IDVG was calculated from plasma decay curves using the one-compartment model by assessing plasma values that were increased above preinjection values between 3 and 7 min postinjection, as described in our previous reports for adults [7]. Duplicate measurements of plasma glucose were made. Coefficients of variation for repeated measurements of plasma glucose (range, 55–300 mg/dL) were ≤1.0%.

### Patient management

All children received a continuous infusion of glucose and electrolytes in a conventional fluid solution using an electric pump through an intravenous line that was different from the one used for glucose administration for IDVG determination. Most children who underwent cardiovascular surgery received a continuous infusion of vasoactive drugs such as dopamine combined with dobutamine (up to 10 μg/kg/min), which had been started during surgery and the infusion rate was kept constant during the study.

Controlled mechanical ventilation with a peak airway pressure above positive end-expiratory pressure (10–15 cmH_2_O with a low positive end-expiratory pressure [<3 cm H_2_O]) was used with continuous infusions of midazolam (1–3 mg/h) in most children, at least until the completion of the study on the operative day. Supplemental morphine (1–2 mg) was injected to avoid excess body movement as required. Except on the operative day, mechanical ventilation or heavy sedation was not consistently required, but an infusion of dexmedethomidine (0.2–0.7 μg/kg/h) was administered in most cases. The infusion rate of these fluids, sedatives, and analgesics was kept constant from at least 30 min before and during the study period. None of the patients had a blood glucose concentration >200 mg/dL before glucose injection, and therefore, insulin was not administered throughout the study period.

### Evaluation points

**Figure 1:**
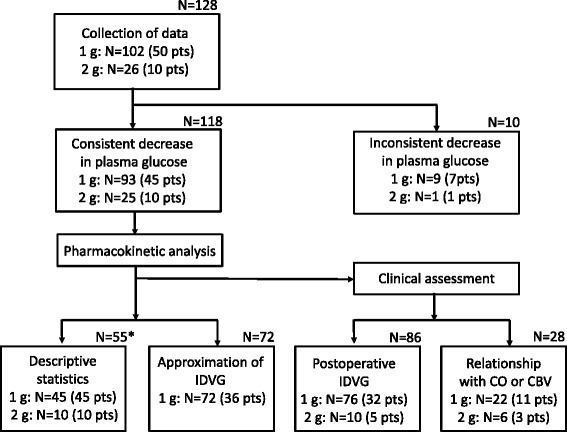
**Flow chart of data collection and evaluation.** *Data for the first measurement from each patient. pts = patients; CO = cardiac output; CBV = circulating blood volume.

Each glucose decay curve was initially examined to determine whether a consistent decrease in incremental plasma glucose concentrations was observed throughout the 7-min period after glucose injection. Ten data sets (7.8%) from eight children failed to show a consistent decrease, which did not permit pharmacokinetic analysis for determining IDVG. Three of these eight children also had data sets associated with a consistent decrease. Thus, the remaining 118 data sets from 55 children, including 8 data sets from the 3 children, were available for analysis in this study (Figure 1). The following pharmacokinetic analysis and clinical assessment were performed.

#### One compartment model

To evaluate whether the one-compartment model could be applied, the following points were assessed: (1) evaluation of the convergence of each glucose decay curve using Akaike’s information criterion (AIC) as reported previously [13] and (2) comparison of IDVG and disappearance rate of incremental plasma glucose levels (Ke-glucose) with our previous data for adult patients.

#### Approximated IDVG

To reduce turnaround time and the amount of blood sampling, approximated IDVG for adults was calculated using the difference between plasma glucose concentrations immediately before and 3-min after glucose injection (5 g) [14]. Approximated IDVG for children (1-point IDVG) was calculated based on the same formula and then adjusted according to the glucose load: 1/5 volume for 1 g of glucose. Thirty-six children with measurements on at least two different days after a 1-g glucose load were examined. The first two data sets from each patient were used for this purpose.

In the early period of this study, Ke-glucose was found to vary markedly between children compared with adults. Moreover, a continued decrease in the elevated plasma glucose concentrations after glucose injection was not consistently observed on some occasions. Accordingly, the use of only one sample at 3 min postinjection for the approximation of IDVG in all children may be inadequate. At least one additional measurement point during the sampling period would be required to overcome this concern. Multiple regression analysis was performed to formulate the revised approximated IDVG for children using plasma glucose concentrations immediately before and at 3 and 5 min postinjection (2-point IDVG). The first 17 of the 36 children were examined for this purpose.

The 1-point IDVG was calculated using the formula for adults [14] as follows:

$$ 1\hbox{-} \mathrm{point}\;\mathrm{IDVG}\;\left(\mathrm{L}\right)=0.2*\left(24.4\;{\mathrm{e}}^{\hbox{-} 0.03\mathrm{x}}+2.7\right) $$

Here, *x* (mg/100 mL) is the incremental plasma glucose concentration at 3 min after 1 g of glucose injection.

The following formula for 2-point IDVG was obtained using multiple regression analysis:

$$ 2\hbox{-} \mathrm{point}\kern0.27em \mathrm{IDVG}\;\left(\mathrm{L}\right)=1\hbox{-} \mathrm{point}\;\mathrm{IDVG}\;\left(\mathrm{L}\right)\hbox{--} 2.5*\left(\mathrm{Diff}\hbox{-} 3,5\; \min \right)+0.35 $$

Here “Diff-3,5 min” represents the difference in the plasma glucose concentration between 3 and 5 min postinjection divided by the plasma concentration at 3 min postinjection. To validate the formula, the relationship between 2-point IDVG and standard IDVG was evaluated in the remaining 19 children.

#### Postoperative IDVG

The time course of perioperative IDVG was evaluated daily in 37 children who underwent cardiovascular surgery (body weight range, 8.0–28.8 kg) who had at least two measurements using either 1 g or 2 g of glucose administration on the operative day and postoperative days 1 and 2.

#### Relationship to CO and CBV

Measurements of CO and CBV were conducted in the second period of this study using pulse dye densitometry (PDD; DDG-2001, Nihon-Koden Co. Ltd., Tokyo, Japan). The first two daily measurements were evaluated in 14 children: 12 who underwent cardiovascular surgery and 2 who underwent non-cardiac surgery. Indocyanine green (ICG) (Daiichi Pharmaceutical Co., Ltd., Tokyo, Japan) was used as the indicator. A percutaneous probe for the detection of the ICG signal was placed at the first or second finger using a nostril probe for adults. Either 5 or 10 mg of ICG was mixed with glucose solution containing 1 or 2 g of glucose, respectively. Immediately after completing the measurements of IDVG and CBV, CO was determined using 1 or 2 mg of ICG. A total of 0.5 mL of normal saline solution was used to flush the central venous line immediately after injection of the indicator. Triplicate CO measurements were made. For reliable measurements, the signal quality index of PDD should account for 1%–5% [15]. The mean signal quality index was 3.6 ± 1.5 (range, 1.1–5.0). Coefficients of variation for triplicate measurements of CO were ≤10.2% for CO.

### Statistical analysis

Unless otherwise stated, data are presented as mean ± SD. IDVG, CO, and CBV were indexed to preoperative basal body weight as required. Statistical analysis was performed with SigmaPlot 12 (Systat Software Inc., San Jose, CA). Perioperative IDVG data were normally distributed, so comparisons were made using repeated measures analysis of variance test, and intergroup comparisons were made using the Holm-Sidak method. Agreement between 1-point IDVG or 2-point IDVG and standard IDVG was assessed by Bland-Altman plots and Pearson’s linear correlation. The latter was also used to determine the relationship between indexed IDVG (IDVGI) and indexed CO (COI) or indexed CBV (CBVI). Spearman’s rank correlation was used to determine the relationship between IDVG and children’s age. *P* <0.05 was considered significant.

## Results

### Characteristics of all recruited children

The mean age, height, and body weight of the group of 60 children comprising 33 male and 27 female were 3.6 ± 2.3 (range, 0.8–8.0) years old, 96 ± 18 (range, 69–135) cm, and 14.4 ± 5.3 (range, 8.0–28.8) kg, respectively. Fifty-three children underwent cardiovascular surgery.

### Basic IDVG data of 55 children

**Figure 2:**
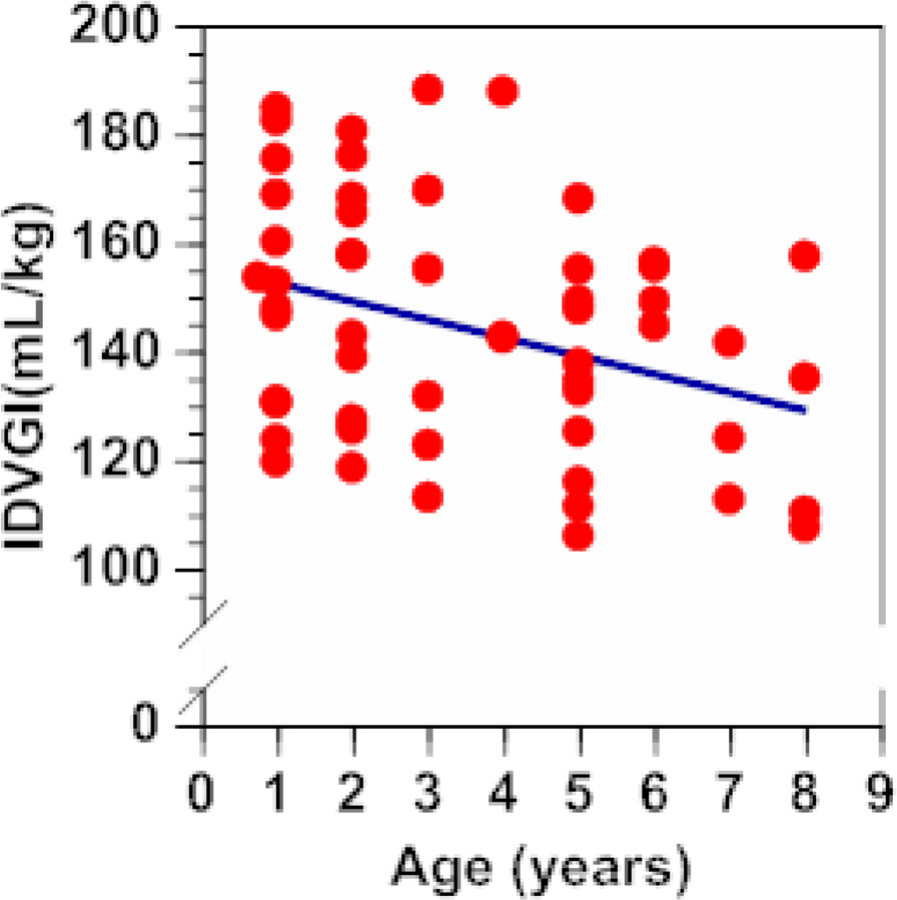
**Age-related changes in indexed IDVG.** Age versus IDVGI: Y = −3.4 X +160 (*r* = −0.309, *n* =55, *p* =0.022). IDVG = initial distribution volume of glucose calculated using the one-compartment model; IDVGI = indexed IDVG based on body weight.

The mean AIC of 118 IDVG measurements derived from the one-compartment model was −30.3 ± 6.0 (range, −49.1 to −18.7), indicating a convergence in each glucose clearance curve. If only the first measurement of IDVG is used in these 55 children, the mean IDVGI was 144 ± 22 mL/kg (range, 106–188 mL/kg). The mean Ke-glucose was 0.094 ± 0.033/min (range, 0.048–0.225/min). There was a weak inverse correlation between IDVGI and age of the tested children (*r* = −0.309, *n* = 55, *p* = 0.022; Figure 2). IDVGI in younger children receiving 1 g of glucose was greater than that in older children receiving 2 g of glucose (*p* = 0.016; Table 1).

**Table 1:** **Patients characteristics**

	**1 g**	**2 g**	***p***
**Glucose load**			
*N* (M/F)	45 (24/21)	10 (4/6)	0.5
Age (years)	3.0 ± 1.9 (2.0, 1.0 to 5.0)	6.4 ± 1.4 (6.5, 5.0 to 8.0)	<0.001
Height (cm)	90 ± 14 (87, 79 to 104)	119 ± 8.9 (119, 111 to 124)	<0.001
Body weight (kg)	12.7 ± 3.7 (12.0, 9.7 to 15.6)	22.0 ± 3.7 (22.3, 19.2 to 24.0)	<0.001
Body surface area (m2)	0.55 ± 0.13 (0.53, 0.45 to 0.67)	0.85 ± 0.1 (0.84, 0.80 to 0.91)	<0.001
Plasma glucose (mg/dL)^a^	106 ± 31 (102, 82 to 128)	120 ± 18 (119, 106 to 131)	0.2
IDVG (L)	1.86 ± 0.53 (1.74, 1.45 to 2.30)	2.83 ± 0.57 (2.68, 2.48 to 3.17)	<0.001
IDVGI (mL/kg)	148 ± 22 (147, 131 to 166)	129 ± 20 (124, 111 to 155)	0.02
Ke-glucose (/min)	0.100 ± 0.034 (0.100, 0.075 to 0.122)	0.068 ± 0.017 (0.065, 0.057 to 0.084)	0.003
AIC	−29.7 ± 5.22 (−28.7, −32.5 to −26.1)	−32.5 ± 7.5 (−33.0, −35.6 to −28.0)	0.18
Surgical procedures or diagnosis			
ASD operation	11	7	
PDA operation	4	1	
Fallot operation	6		
VSD operation	6		
Fontan operation	4		
Bi-directional Glenn operation	4		
Other cardiovascular operation	4	1	
Lung resection	2		
Brain operation	1		
Abdominal operation	1		
Sepsis	1		
Respiratory failure	1		
Major burn		1	

Data are presented as mean ± SD (median, interquatile range) or as number of patients.

^a^Plasma glucose concentration immediately before glucose challenge.

*IDVG* initial distribution volume of glucose, *IDVGI* IDVG based on preoperative body weight, *Ke-glucose* disappearance rate of glucose from plasma, *AIC* Akaike's information criterion, *ASD* atrial septal defect, *PDA* patent ductus arteriosus, *VSD* ventricular septal defect.

### Approximated IDVG

**Figure 3:**
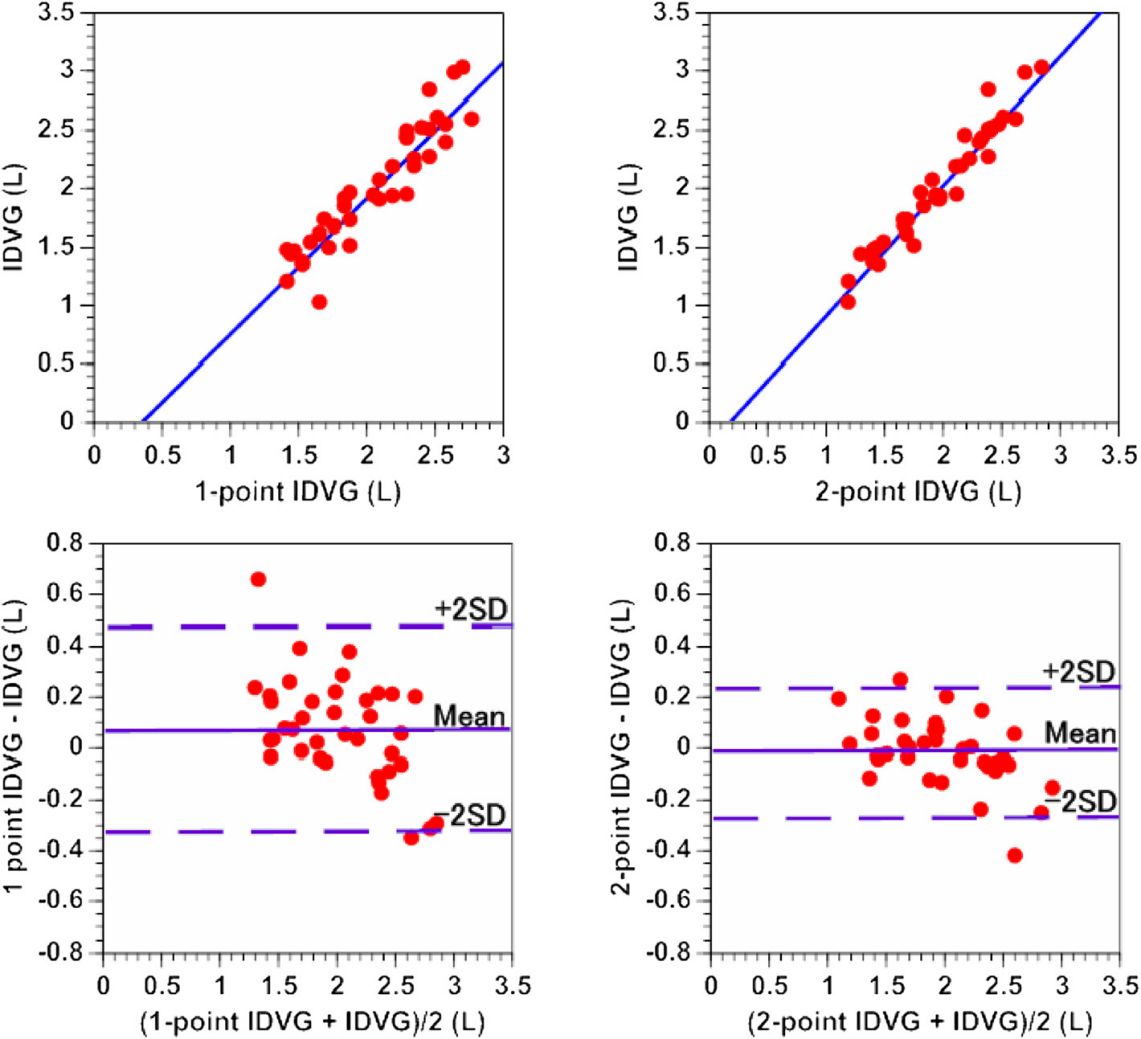
**Relationship between 1-point IDVG and IDVG, and between 2-point IDVG and IDVG.** Top left: 1-point IDVG versus IDVG: Y = 1.2X −0.4 (*r* = 0.929, *n* = 38, *p* <0.001), Top right: 2-point IDVG versus IDVG: Y = 1.1 X −0.2 (*r* = 0.972, *n* = 38, *p* <0.001). Bottom left: Bland-Altman plots for 1-point IDVG versus IDVG (bias and precision, 0.07 ± 0.20 L), bottom right: Bland-Altman plots for 2-point IDVG versus IDVG (bias and precision, −0.02 ± 0.13 L). IDVG = initial distribution volume of glucose calculated using the one-compartment model; 1-point IDVG = approximated IDVG using the incremental plasma glucose value at 3 min postinjection; 2-point IDVG = approximated IDVG using the incremental plasma glucose values at 3 and 5 min postinjection.

Linear correlations were observed between 1-point IDVG and standard IDVG (*r* = 0.929, *n* = 38, *p* <0.001; Figure 3, top left) and between 2-point IDVG and standard IDVG (*r* = 0.972, *n* = 38, *p* <0.001; Figure 3, top right). The bias and precision were smaller between 2-point IDVG and standard IDVG than between 1-point IDVG and standard IDVG (−0.02 ± 0.13 L versus 0.07 ± 0.20 L, p <0.001; Figure 3, bottom left and right).

### Postoperative IDVG

IDVGI on the operative day was 144 ± 21 mL/kg, which increased to 155 ± 24 mL/kg on postoperative day 1 (*p* = 0.011 versus operative day) and to 173 ± 28 mL/kg on postoperative day 2 (*p* = 0.005 versus operative day). Only 12 patients were evaluated on postoperative day 2 (Table 2).

**Table 2:** **Postoperative cardiovascular and fluid volume variables in cardiovascular surgery children**

	**POD0**	**POD1**	**POD2**
Heart rate(bpm)	136 ± 26	111 ± 25*	121 ± 19 (*n* =12)*
SAP (mmHg)	88 ± 20	97 ± 20*	99 ± 26 (*n* =12)*
MAP (mmHg)	58 ± 12	62 ± 13	67 ± 17 (*n* =12)*
CVP (mmHg)	9 ± 5	9 ± 5	10 ± 3 (*n* =12)
COI (mL/kg)	148 ± 35 (*n* =12)	132 ± 49 (*n* =12)	-
CBVI (mL/kg)	70 ± 16 (*n* =11)	77 ± 18 (*n* =10)	-
IDVGI (mL/kg)	144 ± 21	155 ± 24*	173 ± 28 (*n* =12)*

Data are presented as mean ± SD.

*POD* postoperative day, *SAP* systolic arterial pressure, *MAP* mean arterial pressure; *CVP* central venous pressure, *COI* indexed cardiac output based on preoperative body weight; *CBVI* indexed circulating blood volume based on preoperative body weight; *IDVGI* indexed initial distribution volume of glucose based on preoperative body weight.

*p <0.05 compared with POD0.

### Relationship with CO or CBV

**Figure 4:**
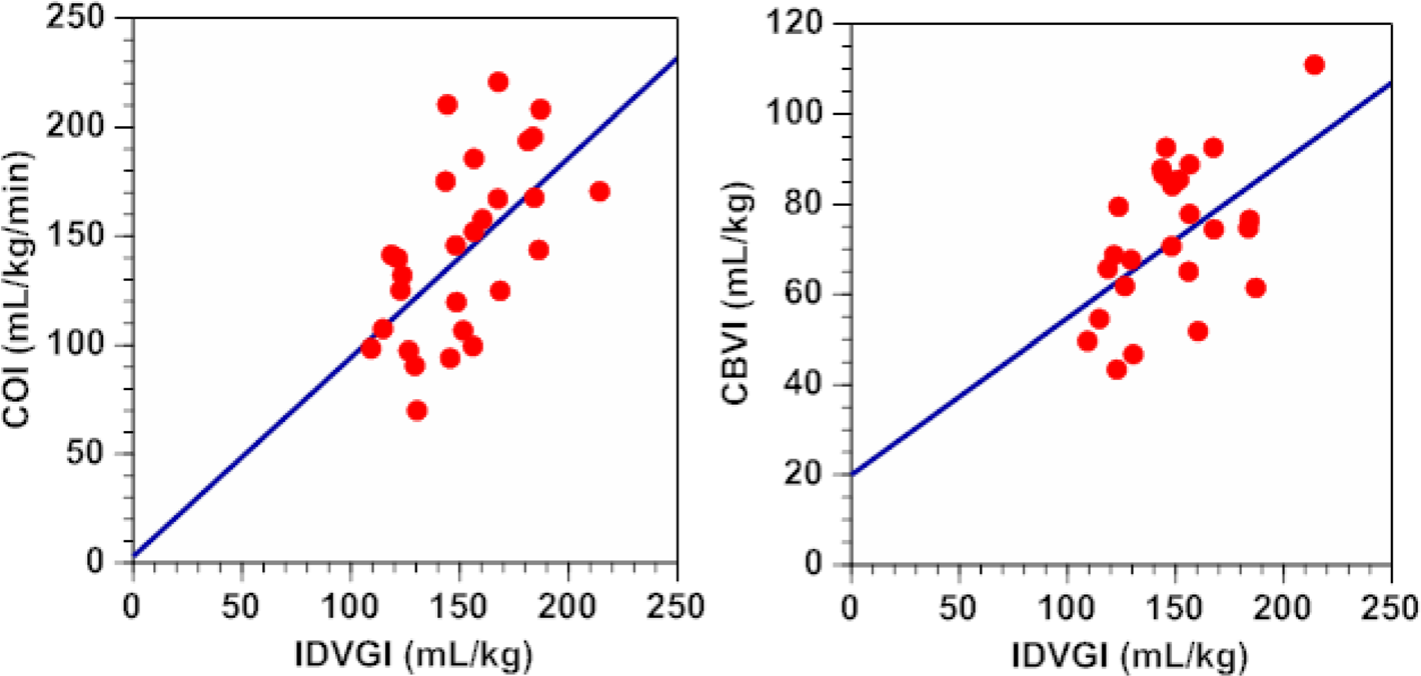
**Relationship between indexed IDVG and indexed cardiac output, and between IDVG and indexed circulating blood volume.** Left: IDVGI versus COI: Y = 0.92 X +3.4 (*r* = 0.588, *n* = 28, *p* =0.00099), right: IDVGI versus CBVI: Y =0.35 X +20 (*r* = 0.547, *n* = 25, *p* = 0.0047). IDVGI = initial distribution volume of glucose calculated using the one-compartment model, IDVGI = indexed IDVG based on body weight, COI = indexed cardiac output based on body weight, CBVI = indexed circulating blood volume based on body weight.

A linear correlation was observed between IDVGI and COI (*r* = 0.588, *n* = 28, *p* = 0.00099; Figure 4, left). Twenty-five measurements were performed for the IDVG and CBV study, since either inappropriate injection of ICG or displacement of the percutaneous probe occurred on three occasions. A linear correlation was observed between IDVGI and CBVI (*r* = 0.547, *n* = 25, *p* = 0.0047; Figure 4, right).

## Discussion

Surprisingly, 7.8% of measurement data did not show a consistent decrease in plasma glucose levels 3 min after glucose administration in the early period of the study. We eventually found that even a small degree of body movement during measurement may cause undesirable plasma glucose changes in children; this phenomenon was not observed in our IDVG study on adults [6]. Therefore, to obtain reliable IDVG data in children, relatively heavy sedation during measurement should be give to prevent body movement-associated glucose fluctuation.

After excluding the abovementioned data, convergence was assumed in each glucose clearance curve (based on the AIC values), which was equivalent to that in adults, even though Ke-glucose was greater than that in our previous report on adults [6]. Most patients in the present study were children who underwent cardiovascular surgery, and they had a mean IDVGI of 144 ± 21 mL/kg on the operative day. The reported mean postoperative IDVG on the operative day for adult patients who underwent cardiac surgery was approximately 106 mL/kg when based on average preoperative body weight and body surface area [16]. This suggests that the postoperative IDVG based on body weight in children is greater. However, IDVG on postoperative days 1 and 2 was increased compared with that on the operative day in both children and adults undergoing cardiovascular surgery.

Moreover, a significant decrease in ECF volume occurs during the first year of life, followed by a smaller decrease later in childhood, from 27.4% of body weight in the first 6–12 months to 22.0% between 5–10 years of age [17]. In this study, IDVGI in children around 3 years old was greater than IDVGI in children around 6 years old (148 and 129 mL/kg, respectively). A weak inverse correlation was also observed between IDVGI and children’s age, which is consistent with age-related physiological change. However, further studies are required to determine the normal IDVGI in children.

We preliminarily checked each approximated IDVG by adding one more sampling point during the 7-min postinjection period other than the 3-min postinjection sampling point to formulate 2-point IDVG. The bias and precision between each approximated IDVG and standard IDVG calculated by the one-compartment model were smallest using the 5-min postinjection sampling point. Consequently, the 5-min postinjection sampling was selected as 2-point IDVG in this study. Evaluation of the data showed that the difference between 2-point IDVG and standard IDVG was smaller than that between 1-point IDVG min and standard IDVG, supporting that 2-point IDVG is preferable to 1-point IDVG in children.

The use of PDD to obtain reliable pulse waveforms in small children and neonates can be difficult [18]. In the early period of this study when using a nostril probe attached to the wing of the nose or the corner of the mouth, the signal quality was very poor and thus reliable data could not be obtained [15]. Subsequently, in this study, the site of probe placement was changed to the index or middle finger. Although a nostril probe for adults was used on children’s fingers, PDD is able to detect the blood dye concentration regardless of the detection site as long as a suitable probe is available [19], so the use of a nostril probe should not be problematic.

The median coefficients of variation of the three consecutive CO derived from PDD (CO-PDD) in this study was 5.4%, which was exactly the same as that in adult patients after cardiac surgery [20] in which measured changes in CO were 81% concordant (i.e., <1 L/min different) between PDD and pulmonary artery catheter (PAC) thermodilution methods in adults. Similarly, Imai et al. [19] reported that the accuracy of CO-PDD was good when compared with the PAC-thermodilution method, as indicated by a mean bias of 0.16 ± 0.80 L/min. The mean coefficient of variation of CO-PDD calculated using their data was 7.8%. However, several other studies reported that CO-PDD was less accurate than thermodilution CO, although there was no information on the repeatability of CO-PDD [21-23]. Presumably, the accuracy of CO-PDD is similar to the thermodilution of CO if the quality of the ICG signal and the repeatability of PDD measurements are satisfactory.

Although CO depends on cardiac preload based on the Frank-Starling relationship, an excessive increase in cardiac preload, a decrease of myocardial contractility, and changes in cardiac afterload may also have a significant impact on the relationship between IDVG and CO particularly after cardiovascular surgery. In this study, a moderate linear correlation was observed between IDVGI and COI (*r* = 0.588). This correlation was comparable to that of our adult cardiac surgery patients (*r* = 0.49) [16].

The moderate linear correlation between IDVGI and CBVI in the present study (*r* = 0.547) is similar to that between IDVG and plasma volume derived from the ICG dilution method after adult cardiac surgery without body weight correction (*r* = 0.68) [24], supporting the hypothesis that IDVG in children reflects the central ECF volume status.

### Limitations

In addition to body movement-associated glucose fluctuation during the measurement, there are several limitations in this study. First, most of the studied children had undergone cardiovascular surgery for congenital heart disease. Therefore, the basic and optimal fluid volume status in these children most likely differed from those of the normal children. Accordingly, IDVG may have varied and reflected the underlying pathophysiology in each child. Second, the measurement of IDVG in this study was performed in relatively stable hemodynamic states. No data were obtained during hemodynamically unstable states such as apparent heart failure and/or hypovolemia, where fluid management based on proper assessment of fluid volume state is crucial. Third, the sample size was too small to convincingly conclude that age-related changes exist in the measurement of IDVG.

## Conclusions

IDVG is a potential clinically relevant marker of fluid volume management, even though body movement-associated glucose fluctuation is a major limitation of this method. IDVG in children can be approximated using two, rather than one, incremental plasma glucose concentrations.

## Abbreviations

AIC: Akaike’s information criterion
CBV: circulating blood volume
CBVI: indexed circulating blood volume
CI: cardiac index
CO: cardiac output
COI: indexed cardiac output
CO-PDD: cardiac output derived from pulse dye densitometry
CVP: central venous pressure
ECF: extracellular fluid
ICG: indocyanine green
ICU: intensive care unit
IDVG: initial distribution volume of glucose
IDVGI: indexed initial distribution volume of glucose
Ke-glucose: Disappearance rate of plasma glucose
PAC: Pulmonary artery catheter
PDD: Pulse dye densitometry
1-point IDVG: Approximated initial distribution volume of glucose calculated by one incremental plasma glucose level at 3 min postinjection
2-point IDVG: Approximated initial distribution volume of glucose calculated by two incremental plasma glucose levels at 3 and 5 min postinjection
